# Hormones, heat, and health: a comprehensive review of sex-based differences in brown and beige fat biology

**DOI:** 10.1186/s13293-025-00787-4

**Published:** 2025-12-31

**Authors:** Chikkamagaluru Gopalakrishna Shashank, Raga Mandali, Umesh D. Wankhade

**Affiliations:** 1 Arkansas Children’s Nutrition Center, Little Rock, AR 72202 USA; 2 Arkansas Children Nutrition Center, Department of Pediatrics, University of Arkansas for Medical Sciences, Little Rock, 72205 AR USA

**Keywords:** Brown fat, Beige fat, UCP1, Sexual dimorphism, Metabolic health, Obesity, Menopause, PCOS, Thermogenic adipocytes

## Abstract

This review takes a close look at the biology of brown and beige fat, not just as thermogenic tissues, but as active metabolic organs influenced by sex, hormones, age, and even environment. Brown adipose tissue (BAT) and beige adipocytes differ in their origins, gene expression, and regulation. These differences are especially relevant when considering how they behave in males and females. Across both animal and human studies, females show higher BAT volume and more efficient thermogenic activity. Estrogen, acting mainly through estrogen receptor alpha (ERα), increases uncoupling protein 1(UCP1) expression, promotes mitochondrial biogenesis, and supports the formation of beige fat within white adipose tissue. In contrast, testosterone and glucocorticoids tend to reduce thermogenic gene expression and shift fat storage toward visceral depots, which increases metabolic risk, particularly in men. These hormone-driven effects are not limited to adulthood. Puberty, pregnancy, menopause, and andropause all influence thermogenic capacity in sex-specific ways. We also outline the key signaling pathways behind beiging such as PR domain-containing 16 (PRDM16), Peroxisome proliferator activated receptor gamma coactivator 1-alpha (PGC-1α), and β3-adrenergic signaling and how they interact with sex hormones to shape thermogenic responses. Findings from Positron Emission Tomography with Computed Tomography (PET/CT) imaging, genetic models, and molecular profiling show that beige and brown fat are regulated by distinct mechanisms and developmental cues depending on sex. We also review how BAT activity is linked to a lower risk of type 2 diabetes, cardiovascular disease, and inflammation, particularly in women with obesity. Conditions like Polycystic Ovary Syndrome (PCOS), hormone therapy, and exposure to endocrine-disrupting chemicals further influence BAT function in sex dependent ways. Understanding how brown and beige fat respond differently in men and women to internal and external signals, is critical. These differences have clear implications for developing targeted, more effective strategies to treat obesity and metabolic disease.

## Highlights

- Females consistently show higher UCP1 expression and BAT activity than males across species, influenced by estrogen dependent mitochondrial and sympathetic regulation.
- Hormonal regulation of BAT and beige adipocytes is profoundly sex specific, with estrogen and thyroid hormones enhancing thermogenesis in females; progesterone exerting dose and context dependent effects; androgens and glucocorticoids generally suppressing thermogenic activity, particularly in males.
- Beige adipocytes arise from diverse progenitor populations, including smooth muscle like and PDGFRα + cells, with their activation influenced by environmental and hormonal cues that show sex-dependent variation in beiging efficiency.
- Cold exposure triggers more robust browning responses in females, including greater UCP1 induction, denser sympathetic innervation, and higher beige adipocyte recruitment.
- Environmental endocrine disruptors such as BPA and phthalates impair beiging and promote BAT whitening, with potential sex specific susceptibility due to differential estrogen receptor signaling.

## Background

Obesity is now one of the most critical public health challenges worldwide. In the United States alone, adult obesity (age ≥ 20 years) increased from 30.5% in 1999–2000 to 41.9% in 2020, affecting over 100 million adults [1]. This escalating weight gain is a driving force behind a cascade of chronic diseases, including Type 2 Diabetes Mellitus (T2DM), cardiovascular diseases, and certain cancers, ultimately undermining quality of life and longevity [2–4]. It’s largely driven by an imbalance between energy intake and expenditure, leading to the accumulation of excess energy as triglycerides in adipose tissue. Traditionally viewed as a passive reservoir for fat storage, adipose tissue has emerged as a dynamic endocrine organ that actively regulates metabolic processes through the secretion of adipokines and cytokines [5]. Adipose tissue is classified based on its locations in the body and performs distinct physiological roles: White adipose tissue (WAT), which serves as the body’s primary lipid storage depot, and BAT, a metabolically active tissue specialized in energy dissipation through heat production. BAT achieves this remarkable feat via non-shivering thermogenesis, mediated by the mitochondrial protein, UCP1, effectively transforming surplus caloric energy into heat [6]. BAT’s ability to generate heat makes it a promising way to counteract weight gain, offering a biological furnace to burn surplus calories. In adult men, cold activated BAT can increase daily energy expenditure by 150 to 300 kcal under optimal conditions [7]. While that may not seem dramatic in the context of total daily intake, even modest, sustained increases in energy expenditure can play a meaningful role in long-term weight regulation and metabolic health.

For decades, BAT was believed to be exclusive to infants and hibernating mammals, fading away as humans age [6]. However, a paradigm shift occurred in 2009 when pivotal imaging studies utilizing positron emission tomography-computed tomography (PET-CT) unveiled active BAT deposits in adult humans especially upon cold stimuli [8, 9]. This discovery sparked significant interest in its biological function in adult metabolism and possible role as a counteracting measure to contain the excessive weight gain.

Another crucial discovery enhancing our understanding of adipose biology is the beiging phenomenon, in which a subset of white adipocytes acquires brown-like characteristics. These “beige” adipocytes exhibit an increased mitochondrial density and upregulated thermogenic genes expression, most notably UCP1 [10]. A variety of stimuli, including chronic cold exposure, sympathetic nervous system (SNS) activation, and endocrine factors such as irisin and fibroblast growth factor 21 (FGF21), can drive this phenotypic transition [11]. By boosting metabolic flux and energy expenditure, beige adipocytes hold promise as a therapeutic target for obesity, potentially complementing the thermogenic activities of classical BAT. The prospect of harnessing both brown and beige fat to combat obesity underscores the intricate regulatory mechanisms of adipose tissue and its capacity for metabolic adaptation.

Moreover, recent evidence suggests that the capacity for beiging, along with the thermogenic potential of BAT, may vary between sexes [12, 13], a distinction with implications for adipose tissue distribution and metabolic regulation. Sexual dimorphism in adipose tissue distribution is well documented, with women typically accumulating fat in the lower body and men predominantly storing fat around the abdomen and internal organs [14]. Women also have a higher overall body fat percentage than men, a difference that is evident since birth and becomes more pronounced during puberty [15, 16]. Females have larger adult brown adipose depots that are not just larger in size but also more efficient in non-shivering thermogenesis [17]. Energy homeostasis clearly differs between the sexes, significantly influencing the development of obesity and related co-morbidities. Sex based differences in BAT activity may play a role in these variations, highlighting the importance of investigating the mechanisms underlying these differences.

**Fig. 1:**
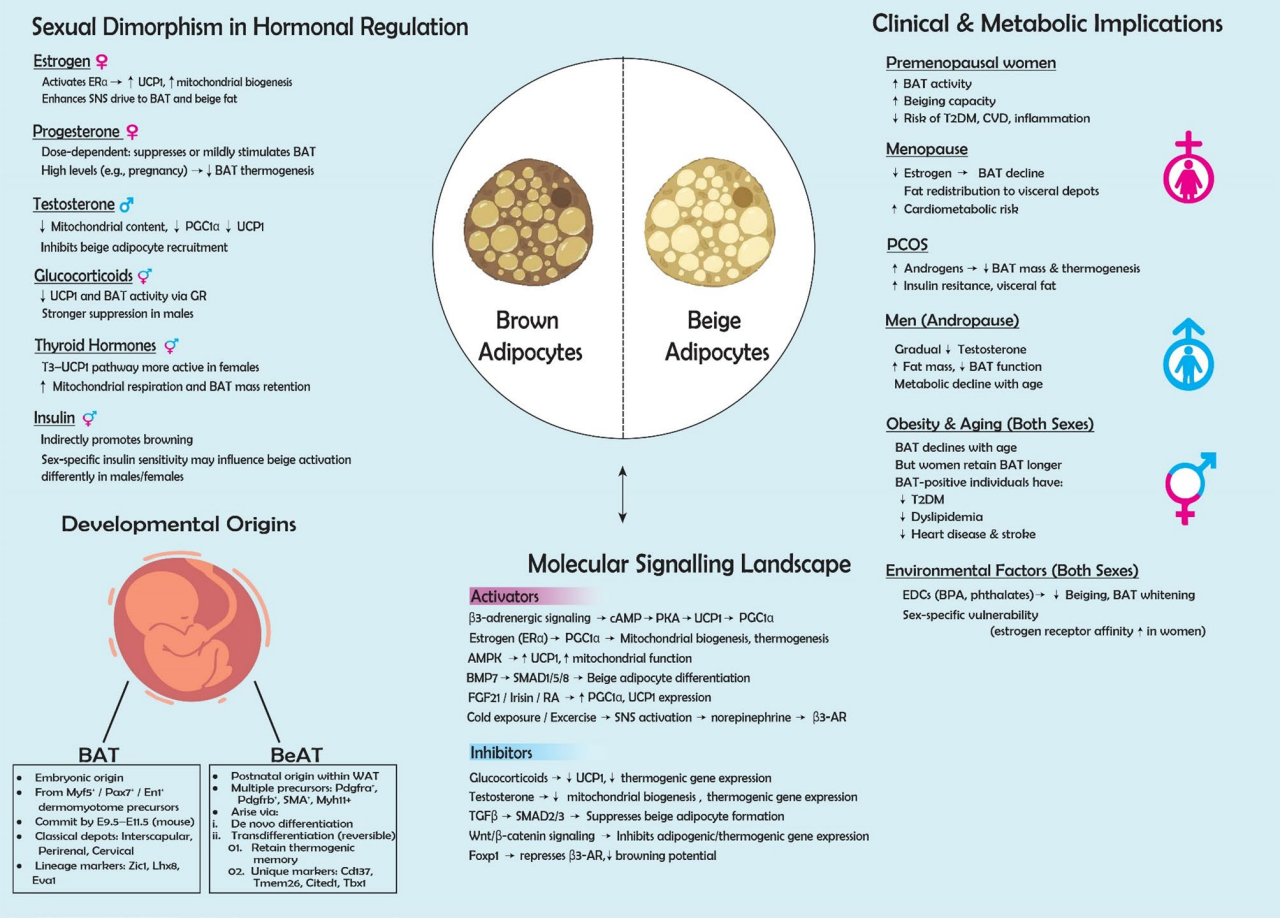
A comprehensive graphical summary integrating key concepts discussed in this review including developmental origins, molecular pathways, hormonal regulators, environmental influences, and sex specific clinical implications. Abbreviations: *ERα*, estrogen receptor alpha; *UCP1*, uncoupling protein 1; *SNS*, sympathetic nervous system; *BAT*, brown adipose tissue; *PGC1α*, peroxisome proliferator-activated receptor gamma coactivator 1-alpha; *GR*, glucocorticoid receptor; *T3*, triiodothyronine; *Myf5+*, myogenic Factor 5; *Pax7+*, paired box transcription factor 7; *En1+*, engrailed homeobox 1; *Zic1*, zinc finger protein of the cerebellum 1; *Lhx8*, LIM homeobox 8; *Eva1*, epithelial V-like antigen 1; *WAT*, white adipose tissue; *Pdgfra+*, platelet-derived growth factor receptor α; *Pdgfrb+*, platelet-derived growth factor receptor beta; *SMA+*, smooth muscle actin; *Myh11+*, myosin heavy chain 11; *Cd137*, cluster of differentiation 137; *Tmem26*, transmembrane protein 26; *Cited1*, Cbp/p300 interacting transactivator with Glu/Asp rich carboxy-terminal domain 1; *Tbx1*, T-box transcription factor 1; *cAMP*, cyclic adenosine monophosphate; *PKA*, protein kinase A; *AMPK*, adenosine 5′-monophosphate -activated protein kinase; *BMP7*, bone morphogenetic protein 7; *SMAD*, mothers against decapentaplegic homolog 1; *FGF21*, fibroblast growth factor 21; *RA*, retinoic acid; *β3 AR*, beta-3 adrenergic receptor; *TGFβ*, transforming growth factor beta receptor; *Wnt*, wingless-related integration site; *Foxp1*, forkhead box P1; *T2DM*, type 2 diabetes mellitus; *CVD*, cardiovascular disease; *EDCs*, endocrine-disrupting chemicals; *BPA*, bisphenol A

Studies highlight that sexual dimorphism plays a pivotal role in BAT activity and beiging, with sex hormones such as estrogen, testosterone, and other hormones serving as crucial regulators [18, 19]. For example, estrogen enhances thermogenesis and energy expenditure, while its decline during menopause contributes to metabolic dysfunction [20, 21]. Despite growing evidence, the underlying mechanisms governing these sex based differences remain unclear, particularly in humans [22]. This review explores the hormonal regulation of BAT, sex specific metabolic implications, and the therapeutic potential of targeting BAT in obesity management. By synthesizing findings from animal models, human studies, and molecular research, we highlight existing gaps and the need for sex specific investigations. Addressing these knowledge gaps may help develop personalized metabolic interventions tailored to both sexes (Fig. 1).

## Brown adipose tissue: structure, function, and regulation

Despite its modest volume in adult humans, BAT exhibits a large impact on systemic metabolism [23], owing to its tightly regulated thermogenic machinery. Evolutionarily conserved across mammals, BAT serves as a rapid-response system to cold and caloric excess, converting chemical energy into heat through highly coordinated molecular pathways [24]. Unlike WAT, BAT is designed for energy expenditure, driven by a unique set of anatomical, cellular, and mitochondrial features [25]. Recent advances in single-cell sequencing, imaging, and metabolic flux have uncovered the dynamic complexity of BAT, highlighting its role not only in thermogenesis but also in lipid clearance, glucose homeostasis, and endocrine crosstalk with peripheral tissues [26].

At the cellular level, brown adipocytes are morphologically and functionally distinct from their white counterparts. BAT is composed of multilocular adipocytes, characterized by numerous small lipid droplets and a dense population of mitochondria enriched with UCP1, which enables its thermogenic function [27]. These mitochondria are structurally adapted for oxidative metabolism, possessing a high surface area and a rich content of iron containing respiratory complexes, which contribute to the tissue’s characteristic brown coloration [28]. In contrast, white adipocytes are unilocular, containing a single large lipid droplet with sparse mitochondria, optimized for long term energy storage in the form of triglycerides. WAT is primarily responsible for lipid accumulation and release during energy demand, while BAT continuously oxidizes substrates to produce heat, particularly under sympathetic stimulation [29].

Recent studies have shown that lipid droplet morphology is functionally linked to the metabolic roles of each adipose type. This morphological difference is regulated by distinct isoforms of fat specific protein 27 (FSP27): FSP27α promotes unilocular droplet formation in WAT, while BAT expresses FSP27β, which inhibits cell death inducing DNA fragmentation factor alpha-like effector A (CideA) homodimerization and facilitates the formation of small, multilocular droplets, promoting efficient lipolysis and fatty acid oxidation [30].

Beyond structural adaptations, BAT and WAT are transcriptionally divergent. BAT exhibits a thermogenic gene program characterized by high expression of UCP1, PGC-1α, PRDM16, and iodothyronine deiodinase 2 (DIO2), while WAT lacks these thermogenic regulators [31]. PRDM16 is particularly crucial in establishing brown adipocyte identity; its overexpression in myoblasts induces a brown fat phenotype, while its absence in precursors leads to muscle like characteristics [32]. Other markers, such as zinc finger protein of the cerebellum 1 (ZIC1), preferentially expressed in BAT, further define brown adipocyte lineage and function [33]. Moreover, BAT demonstrates elevated glyceroneogenesis activity due to increased phosphoenolpyruvate carboxykinase (PEPCK) levels, supporting fatty acid cycling and thermogenesis [34]. Importantly, the thermogenic phenotype is plastic, under specific stimuli such as chronic cold exposure or β-adrenergic signaling, WAT can undergo “browning,” acquiring BAT like features including mitochondrial biogenesis and UCP1 induction. This adaptive transformation is regulated by endocrine factors like irisin and FGF21 and represents a promising therapeutic avenue for increasing energy expenditure and combating obesity [35].

This process is driven by UCP1, a mitochondrial protein that transports protons (H⁺) from the intermembrane space back into the mitochondrial matrix, bypassing adenosine triphosphate (ATP) synthase. This dissipates the proton motive force used by ATP synthase, increases respiratory chain activity, and converts energy from substrate oxidation into heat [36]. BAT thermogenesis is activated by sympathetic nervous system (SNS) stimulation. Upon cold exposure or β3-adrenergic activation, norepinephrine binds to β3-adrenergic receptors, triggering a cAMP–PKA signaling cascade that enhances lipolysis and upregulates UCP1 expression [37]. The resulting free fatty acids (FFAs) serve as both fuel and UCP1 activators.

In addition to UCP1, UCP1-independent thermogenic pathways such as futile creatine cycling [38, 39] and Sarco(endo)plasmic Reticulum Calcium ATPase (SERCA) mediated calcium cycling also contribute to thermogenesis, especially in beige adipocytes or prolonged activation states [40]. As mentioned above, thermogenic efficiency is further regulated by PGC-1α, thyroid hormones, and endocrine factors like FGF21, irisin, and natriuretic peptides, which enhance mitochondrial function and promote WAT browning. Collectively, these pathways enable BAT to act as a dynamic regulator of energy balance and a promising target for treating metabolic disease.

## Beige adipocytes and the Beiging process

Beige adipocytes also termed “brite” or “inducible brown like” cells have emerged as a unique thermogenic cell type embedded within WAT, particularly subcutaneous depots, in response to environmental stimuli such as chronic cold exposure and β3-adrenergic stimulation [41]. Unlike classical brown adipocytes, which are present in dedicated depots and exhibit high basal expression of UCP1, beige adipocytes are typically quiescent under basal conditions and acquire thermogenic capacity only upon activation [10]. This inducibility, combined with depot specific prevalence and functional plasticity, distinguishes them as a separate entity within the adipose tissue hierarchy.

Histologically, beige adipocytes resemble brown adipocytes, with multilocular lipid droplets and dense mitochondrial content [42]. However, classical brown adipocytes originate during embryogenesis from a subset of dermomyotomal precursors marked by the expression of transcription factors such as paired box transcription factor 7 (Pax7), Engrailed-1 (En1), and myogenic Factor 5 (Myf5) [43–45], with lineage divergence from the somite lineage occurring between embryonic days E9.5 and E11.5 in mice [44]. In contrast, beige adipocytes develop postnatally within WAT, and their origins are notably more diverse and context dependent [42]. Beige adipocytes can be recruited in response to various external and physiological stimuli, including chronic cold exposure, β-adrenergic stimulation, exercise, and certain pharmacological agents [42]. Lineage tracing studies in mice have shown that beige adipocytes can arise from multiple progenitor pools marked by smooth muscle actin (Sma), myosin heavy chain 11 (Myh11), Pdgfrα, and Pdgfrβ [46–49], and in some anatomical depots, also from Pax3 or Myf5 expressing precursors [50]. These findings suggest a complex and depot specific developmental program for beige adipocytes. Moreover, clonal analyses demonstrate that beige adipocyte precursors are molecularly distinct from white adipocyte progenitors in both mice [51] and humans [52, 53], further supporting the concept that beige adipocytes represent a heterogeneous population with discrete origins. This diversity in origin suggests that beige adipocytes are not all the same; rather, they likely include several subtypes that develop in specific fat depots and respond differently depending on the type of stimulus.

Mechanistically, beige adipocytes can arise either through de novo differentiation of precursor cells within the stromal vascular fraction or via transdifferentiation of mature white adipocytes [54]. The relative contribution of each pathway appears to depend on the nature of the beiging stimulus [55]. For example, chronic cold exposure primarily drives differentiation from resident progenitors, while β-adrenergic signaling can also induce phenotypic conversion of mature white adipocytes [56]. Notably, this transdifferentiation is reversible upon withdrawal of thermogenic cues, beige adipocytes can undergo “whitening,” characterized by mitochondrial clearance via autophagy and return to a white like phenotype [54, 57]. However, these cells retain a form of “thermogenic memory,” enabling rapid reacquisition of beige features upon re-stimulation [57]. A wide array of transcriptional regulators, signaling cascades, and hormonal cues have been implicated in orchestrating the recruitment and thermogenic programming of beige adipocytes (Table 1).

**Table 1:** Key pathways and factors involved in the Beiging process of white adipose tissue

Category	Pathway/Factor	Role in Beiging	Reference
Adrenergic & Canonical Signaling	β3-Adrenergic Signaling	Cold or β3 agonists activate β3-AR → cAMP → PKA → UCP1/PGC1α expression.	[58]
	cAMP-PKA pathway	Canonical β-adrenergic pathway for UCP1 induction and beiging.	[37]
	PKA-mTORC1 signaling	Necessary for cold induced ‘browning’ of WAT	[59]
Developmental & Differentiation Pathways	Hedgehog and Notch Pathway	Sequentially activated during beige adipogenesis; regulate differentiation of progenitors into beige adipocytes	[60]
	PI3K-Akt, MAPK, PPARγ	Signal pathways involved in beige adipocyte precursor commitment and thermogenic programming.	[60–63]
	BMP7/TGFβ Signaling	BMP7 supports beiging by activating SMAD1/5/8, while TGFβ activates SMAD2/3 to suppress thermogenic gene expression and beige adipocyte formation	[37]
	Wnt/β-catenin signaling	Suppresses adipogenic commitment and thermogenic gene induction; its inhibition enhances browning in WAT.	[64]
Core Regulators & Transcriptional Control	PGC1α, PRDM16, UCP1	Core regulators of beige adipogenesis and mitochondrial biogenesis.	[65–67]
	Foxp1	Suppresses beiging and thermogenesis by repressing β₃-AR transcription; adipose specific deletion increases β₃-AR, UCP1 expression, and browning.	[68]
	CDK5 inhibition (roscovitine)	Inhibition enhances UCP1 expression and generates distinct beige adipocytes.	[69]
Metabolic & Stress Sensors	Calcium Signaling	Regulates late stage differentiation and mitochondrial function via intracellular Ca²⁺.	[60, 70]
	AMPK	Enhances UCP1 expression, mitochondrial biogenesis, and cold induced beiging; essential for beige function.	[71]
	GDF15	Stress response cytokine promoting thermogenesis and WAT browning.	[72]
Endocrine & Hormonal Cues	Irisin (FNDC5)	Exercise induced myokine; induces beiging via PGC1α and UCP1.	[73]
	Meteorin like (Metrnl)	Induces eosinophil mediated activation of M2 macrophages for beiging.	[74]
	Retinoic Acid	Stimulates UCP1, CideA and Cox7a1 in adipocytes; enhances browning.	[75]
	Neuregulin 4	Enhances energy expenditure and supports beige adipocyte activity.	[76]

*β3-AR*, β3-adrenergic receptor; *AMPK*, AMP-activated protein kinase; *BMP7*, bone morphogenetic protein 7; *CDK5*, cyclin-dependent kinase 5; *CideA*, cell death-inducing DFFA-like effector a; *Cox7a1*, cytochrome c oxidase subunit 7A1; *FGF21*, fibroblast growth factor 21; *FNDC5*, fibronectin type III domain-containing protein 5; *GDF15*, growth differentiation factor 15; *MAPK*, mitogen-activated protein kinase; *Metrnl*, meteorin-like protein; *mTORC1*, mechanistic target of rapamycin complex 1; *PGC1α*, peroxisome proliferator-activated receptor gamma coactivator 1-alpha; *PKA*, protein kinase A; *PPARγ*, peroxisome proliferator-activated receptor gamma; *PRDM16*, PR domain containing 16; *SMAD*, mothers against decapentaplegic homolog; *TGFβ*, transforming growth factor beta; *UCP1*, uncoupling protein 1; *WAT*, white adipose tissue

Given their diverse developmental origins, beige adipocytes are regulated by a wide range of transcriptional, hormonal, and environmental signals that determine their recruitment and thermogenic activation. Although beige and classical brown adipocytes share core thermogenic genes like UCP1, CideA, and PGC-1α upon activation, beige adipocytes do not express classical brown markers such as Zic1, LIM homeobox 8 (Lhx8), or epithelial V-like antigen 1 (Eva1) [10]. Instead, they exhibit a unique molecular signature, including genes like Cd137, transmembrane protein 26 (Tmem26), Cited1, and Tbx1 [51, 77, 78], further distinguishing them as a separate thermogenic lineage. Transcriptional regulators such as Proliferator-Activated Receptor gamma (PPARγ), PRDM16, and PGC-1α are central to the beiging program, driving mitochondrial biogenesis and thermogenic gene expression [65, 66]. PRDM16, for instance, interacts with co-regulators like euchromatic histone lysine methyltransferase 1 (EHMT1) and Transducin-like enhancer of split 3 (TLE3) to switch white adipocyte identity toward a beige phenotype [79, 80]. Interestingly, beiging is not solely reliant on UCP1. Emerging evidence highlights alternative mechanisms, such as the Peptidase M20 domain-containing 1 (PM20D1) pathway, which generates N-acyl amino acids that act as endogenous mitochondrial uncouplers, boosting energy expenditure independently of UCP1 [81]. Hormones and cytokines also play critical roles in regulating beige adipocyte recruitment and thermogenic function.

Irisin and FGF21 promote beige adipocyte formation and metabolic benefits [81], while cold induced cytokines like IL-4 and IL-13 activate macrophages that release catecholamines, thereby encouraging beige adipocyte recruitment from WAT progenitors [82]. Altogether, these findings underscore that beige adipocytes are not only developmentally distinct but also highly adaptable, responding to a wide range of physiological signals to maintain energy balance.

## Sexual dimorphism in brown fat and Beiging

Sexual dimorphism profoundly influences adipose tissue biology, including differences in fat distribution, BAT activity, and susceptibility to metabolic disorders. These variations are largely orchestrated by sex hormones and manifest in both anatomical and functional disparities between males and females. Understanding the hormonal and molecular underpinnings of these differences is essential for developing sex specific strategies in obesity and metabolic disease treatment.

### Fat distribution and Sex-Specific differences

Sexual dimorphism, which encompasses differences in size, appearance, and physiology between males and females of the same species [83], is a fundamental biological concept often shaped by evolutionary pressures [83]. Body fat distribution in humans is sexually dimorphic, with premenopausal women storing more subcutaneous adipose tissue (SCAT) in the gluteofemoral region [84], characterized by adipocyte hyperplasia [85], whereas men preferentially accumulate visceral adipose tissue (VAT) in the abdominal area, where adipocyte hypertrophy is more prominent [85, 86]. Following menopause, women exhibit a marked redistribution of fat toward VAT, similar to men [14], which correlates with a higher risk of insulin resistance and cardiovascular disease. These deposition patterns are largely governed by sex hormones. Estrogens promote fat storage in SCAT via ERα-mediated pathways, enhancing lipogenic gene expression in gluteofemoral depots [87]. Conversely, testosterone contributes to visceral fat accumulation by inhibiting SCAT expandability and promoting lipolysis in VAT through activation of androgen receptors [88]. The hormonal transition accompanying menopause, characterized by declining estrogen, further drives fat redistribution toward central depots and contributes to the onset of metabolic dysregulation [89].

In the context of thermogenic fat, sexual dimorphism is evident in both BAT and beige adipose tissue. Premenopausal women have higher BAT activity than men, with a reported prevalence of 44.6% versus 35.9% [90], a difference attributed in large part to the influence of sex hormones. Estrogen, for instance, promotes the expression of thermogenic genes such as UCP1 in WAT, thereby enhancing the beiging potential in females [18, 91]. In contrast, testosterone has been shown to reduce mitochondrial number and respiratory capacity in beige adipocytes [92]. These adaptations provide females with metabolic advantages, including improved lipid oxidation and protection against diet induced obesity. In addition, imaging studies using Fluorodeoxyglucose (FDG) PET/CT have shown that females possess approximately twice as much BAT in the cervical supraclavicular region as males, with a distribution ratio of 2:1 [8], possibly due to greater cold sensitivity [93].

In human studies, BAT has consistently been identified in defined anatomical regions, including the cervical, supraclavicular, mediastinal, and perirenal areas through ^18F-FDG PET-CT imaging [8, 9]. While these studies reported a higher prevalence of BAT in women compared to men, they did not provide sex specific differences in the anatomical localization of BAT depots. For example, Cypess et al. [8] identified BAT in 7.5% of women and 3.1% of men but described similar distribution sites across sexes without depot level comparison. Likewise, van Marken Lichtenbelt et al. [9] noted active BAT mainly in the supraclavicular region in both sexes but did not distinguish depot variation by sex. Regarding beige adipose tissue, studies e.g., by Jespersen et al. [94] showed that deep neck fat depots in adults contain multilocular, UCP1-positive adipocytes, characteristic of beige or brown like cells; however, the study did not differentiate these depots between males and females. Thus, although BAT and beige adipocytes are clearly present in specific anatomical locations in humans, current evidence does not support the existence of sex based differences in their regional distribution.

### Sex differences in BAT activity and volume

Males and females exhibit distinct patterns in BAT volume, activity, and responsiveness, influenced by hormonal and developmental factors. During childhood and early adolescence, some studies report greater BAT activity in boys. A study found that boys at late pubertal stages had greater BAT volume compared to age matched girls (499 ± 246 cm³ in boys vs. 286 ± 139 cm³ in girls), as measured by PET/CT [95]. MRI-based assessments by Deng et al. [96] further demonstrated that boys aged 9–15 years had increased perfusion coefficients and T2* values in BAT, indicative of higher thermogenic potential. Another study observed an increase in BAT% in boys compared to girls [97]. These differences may be attributed to the stronger association between BAT and muscle volume in boys, reflecting their shared developmental origin from Myf5 + progenitor cells and higher skeletal muscle gains during puberty [32, 98]. Further, Robinson et al. [99] noted that in girls, higher vegetable and protein intake correlated with lower supraclavicular temperatures, implying possible sex specific dietary modulation of BAT. However, contrasting findings were observed by Malpique et al. [100], who reported that prepubertal girls exhibited significantly higher baseline and post cold BAT activation, as measured by supraclavicular skin temperature, compared to boys (*P* ≤ 0.01). BAT activity increased more in appropriate for gestational age (AGA) girls than in AGA boys, but this dimorphism was absent in small for gestational age (SGA) children, suggesting that fetal growth may influence how BAT differs between sexes. These findings underscore the dynamic nature of BAT during childhood and adolescence, with sexual dimorphism potentially influenced by pubertal stage, body composition, and perinatal factors.

Though BAT activity varies during childhood, sexual dimorphism becomes more defined in adulthood, particularly between premenopausal women, menopausal women, and men. A study by Pfannenberg et al. [101] using ^18^F-FDG PET/CT scans reported that women exhibited significantly greater BAT mass and activity than men, despite comparable BMI, with a sharper age related decline observed in men. Controlled cold exposure studies yield mixed findings. Chen et al. [102] found increased energy expenditure in premenopausal women during cold exposure (19 °C) compared to men, though 18F-FDG uptake remained similar across sexes. Likewise, Fletcher et al. [103] reported no sex based differences in BAT volume or glucose uptake in young adults, but noted dorsocervical BAT presence in half of the women, compared to just one man. Martinez-Tellez et al. [104] further observed significantly greater active dorsocervical BAT in young women than men, supporting a female predominance in this specific depot. Herz et al. [90] showed that while BAT volumes did not differ significantly between men and premenopausal women, thermogenic activity was higher in women and positively associated with circulating estradiol. They also observed variations in women’s thermogenesis across the menstrual cycle, with higher BAT activity in the follicular phase compared to the luteal phase, where levels were similar to those in men. In another study, BAT activity did not significantly differ between the follicular and luteal menstrual phases in premenopausal women. However, during the follicular phase, BAT activity was positively correlated with plasma FGF21 and 17β-estradiol levels, suggesting estrogen dependent FGF21 modulation of thermogenesis [105]. While men may show comparable or even greater absolute BAT volume due to larger body size, when normalized to body mass or surface area, these differences disappear, and functional activity is often lower than that of women [103]. Collectively, these findings imply that men are less likely to maintain metabolically active BAT with age, potentially increasing susceptibility to metabolic dysregulation.

Sexual dimorphism is evident in beige adipocytes, with females showing a greater capacity for WAT browning. In mice, loss of ERα impaired beige fat formation and worsened metabolic health. Interestingly, treatment with CL 316,243, a selective β3-adrenergic receptor agonist commonly used to pharmacologically induce thermogenesis and WAT browning, rescued beiging and improved insulin sensitivity even in ERα-deficient females, though wild type females still showed more robust multilocular beige cells. Additionally, CL 316,243 increased ERβ expression in WAT, suggesting a compensatory pathway supporting beiging [106]. Complementing these findings, recent work has shown that genetic ablation of cyclin dependent kinase inhibitor 2 A (Cdkn2a), a cell cycle regulator, promotes the long term maintenance of beige adipocytes in both male and female mice after withdrawal of cold exposure. Cdkn2a deficiency enhanced UCP1 expression and preserved thermogenic gene profiles over time, preventing beige to white adipocyte transition. Notably, this effect occurred equally in males and females, suggesting that beige fat maintenance may also be governed by sex independent molecular mechanisms alongside hormone dependent ones [107].

### Role of sex hormones in BAT regulation and thermogenesis

Given the evident sexual dimorphism in BAT, it is essential to investigate the hormonal drivers behind these disparities, particularly in relation to BAT function. Hormones such as estrogen, progesterone, testosterone, and glucocorticoids play pivotal roles in modulating BAT activity. For instance, estrogens are fundamental in regulating BAT activity and differentiation [18]. Ovariectomy leads to decreased thermogenic activity and UCP1 mRNA expression, but administration of Estradiol (E2) can restore these levels in both protein and mRNA levels [21, 108]. A recent study has also provided the first evidence in humans that the loss of estrogen and ovarian function reduces BAT oxidative metabolism and glucose uptake in women, independent of age [109]. The reduction in thermogenic activity post ovariectomy is likely linked to lower levels of Bone Morphogenetic Protein 8B (BMP8B), an adipokine crucial for adaptive thermogenesis [110].

Estrogen, primarily through ERα, promotes BAT proliferation and UCP1 expression [111]. In females with BAT specific ERα deficiency, reductions in UCP1 expression, BAT browning, body temperature, and metabolic response to cold are observed, underscoring estrogens’ role via ERα in BAT function regulation [112]. Conversely, In male mice, increasing adipose tissue aromatase activity enhanced local estrogen production, which improved insulin sensitivity, reduced inflammation in both white and brown adipose depots, and modulated metabolic gene expression, indicating that estrogens play an active regulatory role in male adipose tissue, including BAT [113]. Nonetheless, ERβ-selective ligands have demonstrated anti-obesity effects, reducing lipid accumulation in BAT and enhancing mitochondrial biogenesis marker expression in both BAT and WAT in high fat diet fed mice across both sexes [114, 115]. Moreover, estrogens boost BAT activity and thermogenesis by directly stimulating brown adipocytes and activating the sympathetic nervous system through hypothalamic circuits [116]. Administration of estradiol in female rats elevates UCP1 levels in BAT, along with increased supraclavicular and core body temperatures, highlighting the central role of estrogens in BAT activation [21]. Therefore, estrogens enhance BAT activity and thermogenesis via both direct actions and indirect pathways mediated by the sympathetic nervous system.

In addition to BAT, sexual dimorphism extends to beige adipocytes, with females exhibiting a greater beiging capacity largely driven by estrogen through ERα. In aging male mice, estrogen administration markedly enhanced cold induced iWAT beiging, upregulated thermogenic genes (UCP1, CideA, cytochrome c oxidase subunit 8B (Cox8b)), improved glucose metabolism, and increased core body temperature and energy expenditure [117]. Both in vitro and in vivo studies further confirm the essential role of ERα in beige adipocyte formation. ERα activation significantly increased UCP1 and thermogenic gene expression, whereas ERα knockdown impaired estrogen and β3-adrenergic induced beiging. In vivo, ERα-deficient (ERKO) male mice displayed reduced UCP1 expression and glucose uptake in BAT, while adipose specific ERα overexpression restored beige morphology in female ERKO mice. Mechanistically, ERα activation promoted ATGL mediated lipolysis and AMPK phosphorylation, key events for thermogenic activation. Notably, membrane localized ERα exerted a stronger effect on UCP-1 induction than nuclear ERα, emphasizing the importance of membrane initiated ERα signaling in driving beige adipogenesis [111].

Research on the effects of progesterone (P_4_) on BAT has yielded inconsistent results. Some studies indicate that P_4_ decreases basal UCP1 mRNA expression and suppresses norepinephrine stimulated UCP1 expression and lipolysis in cultured brown adipocytes of both sexes [118]. Conversely, other research has noted P_4_ at low doses stimulates these parameters in vitro, suggesting a dose dependent stimulatory effect on thermogenesis [119]. Elevated P_4_ during pregnancy reduces BAT activity in murines, as seen in decreased mitochondrial content and thermogenic activity, likely conserving energy for fetal development [118, 120]. This inhibitory effect is also observed in P_4_ treated oophorectomised mice [118]. In humans, although direct measures of BAT activity are absent, an increase in supraclavicular temperature during the luteal phase suggests heightened BAT thermogenesis, corresponding with elevated P_4_ levels [121]. While direct studies on beige adipocytes are limited, these findings suggest that P_4_ may contribute to sex specific regulation of beige fat, particularly in females during reproductive states, highlighting an area for future research.

Unlike estrogen, the influence of androgens on BAT activity is more complex. In rodent brown adipocytes (in vitro studies), testosterone was found to inhibit mitochondrial biogenesis, differentiation, and norepinephrine induced lipolysis [119, 122]. Conversely, orchiectomy increased UCP1 expression (60% above baseline) and body temperature in murine BAT [123, 124]. Clinical studies indicate that androgen level changes may have different effects in men and women. Women with PCOS reportedly exhibit lower BAT activity, inferred from reduced supraclavicular skin temperature [125]. In androgen induced PCOS mouse models, excess androgens reduced BAT mRNA expression levels (PGC-1α, UCP1) and other mitochondrial function genes (Dio2, medium-chain acyl-CoA dehydrogenase (Mcad)), possibly leading to lower body temperature [126]. Additionally, in males, aromatase in extragonadal tissues such as adipose tissue and brain provides local estrogen that can act in a paracrine or intracrine manner [127]. Thus, the impact of testosterone may involve ER mediated pathways. For instance, the reduction of white fat mass by testosterone in obese hypogonadal male mice was shown to require ERα in the brain [128]. This suggests that androgens may influence BAT function primarily through central mechanisms, possibly via central estrogen signaling, though further research is needed.

Both BAT and WAT contains glucocorticoid receptors [129]. Excessive levels of glucocorticoids (GC) increase WAT mass and result in weight gain [130]. Conversely, GC have an inhibitory effect on BAT activity in rodent models [131]. GC enhance appetite, stimulate lipolysis, suppress thermogenesis [132, 133] and profoundly suppress norepinephrine induced UCP1 activation in both male and female mice [131, 134]. Similar results were observed in studies involving brown adipocytes (in vitro), as GC reduced the norepinephrine stimulated UCP1 mRNA expression [131, 134]. GC inhibition of BAT is primarily mediated by the glucocorticoid receptor (GR), with GR antagonists reversing this effect on UCP1 mRNA expression [135, 136]. Conversely, GC also interact with the mineralocorticoid receptor (MR), which is expressed in BAT [137]. In female mice, MR deficiency prevents lipid accumulation in BAT under a high fat diet [138], and MR antagonists enhance the browning of white adipose tissue and increase BAT activity and volume in both obese female mice and healthy volunteers (human) [138, 139]. In rodent models, removal of the adrenal glands (adrenalectomy) has been found to enhance BAT thermogenesis and promote weight loss. This effect is likely due to the elimination of glucocorticoid driven suppression of BAT activity via the hypothalamus and can be reversed by administering glucocorticoids [140]. While the influence of this crosstalk on BAT activity remains to be fully elucidated, emerging evidence suggests differential responses in BAT based on sex. For instance, corticosterone treatment has been shown to increase the expression of GR target genes such as FK506 binding protein 5 (Fkbp5) and TSC22 domain family member 3 (Tsc22d3) more significantly in male BAT compared to female BAT. Despite this, the extent of BAT whitening and the reduction in UCP1 mRNA expression appeared consistent across sexes [141]. Notably, another study using a chronic corticosterone dose reported that GC induced suppression of BAT was more pronounced in males than females, highlighting the modulatory role of androgens in sensitizing BAT to GC effects [123].

Thyroid hormones play a pivotal role in regulating energy homeostasis, thermogenesis, and mitochondrial function through their actions on BAT and inducible beige adipocytes. These effects are not only age dependent but also exhibit clear sexual dimorphism. The following synthesis highlights findings from recent studies that explore the interplay between thyroid hormones and adipose tissue biology in male and female models. In a rodent aging model, Valle et al. [142] demonstrated that younger female rats (6 month old) displayed superior BAT thermogenic capacity compared to age matched males. This was evidenced by significantly higher UCP1 content, mitochondrial protein levels, and BAT mass relative to body weight. These sex differences diminished with aging, yet females maintained higher thermogenic capacity until later ages (24 months) compared to males (18 months). Importantly, serum triiodothyronine (T_3_) levels showed strong positive correlations with BAT functional markers, including UCP1 content, COX activity, and mitochondrial respiration, especially in females. This suggests that T_3_ is a key contributor to the observed sex specific BAT thermogenic preservation during aging. Further analysis from the same study revealed that serum T_3_ was initially higher in young female rats than males, though it declined with age, eventually eliminating this gender difference by 18 months. Meanwhile, thyroxine (T_4_) levels were consistently higher in males but also declined with age. These changes were associated with differential mitochondrial functionality, where T_3_ strongly correlated with mitochondrial differentiation markers in females, but only with bulk mass in males [142]. These results collectively support a model where thyroid hormone driven mitochondrial biogenesis and uncoupling activity in BAT are more pronounced and better preserved in females, a phenomenon potentially mediated by sex steroid thyroid hormone crosstalk. In a developmental context, a maternal hyperthyroid model demonstrated that gestational T_3_ exposure led to persistent increases in offspring BAT thermogenesis in both sexes. Offspring from T_3_ treated dams showed elevated interscapular BAT temperatures at postnatal day 5 and again at ≥ 10 weeks of age, regardless of sex. However, no differences were observed in body temperature, oxygen consumption, respiratory quotient, daily energy expenditure, food intake, or resting metabolic rate at 30 °C, indicating that the thyroid‑driven enhancement of BAT activity may occur without systemic metabolic consequences under those conditions [143].

Clinical studies in patients undergoing treatment for Graves’ disease provide evidence of a bidirectional relationship between thyroid hormones and BAT activity. In one investigation, individuals with greater baseline BAT activity demonstrated a larger decline in free T_3_ during the transition from hyperthyroidism to euthyroidism, suggesting that BAT may influence thyroid hormone clearance or tissue responsiveness during recovery. In contrast, another study found that BAT negative individuals exhibited a more robust increase in adiponectin after antithyroid therapy, whereas this effect was blunted in BAT positive individuals. Although neither study looked at sex specific differences, the results point to a meaningful link between BAT activity and how the body adjusts its thyroid hormones and metabolism during recovery from hyperthyroidism [144, 145]. Together, these findings underscore the crucial role of thyroid hormones in maintaining BAT functionality and highlight distinct sex specific responses in thermogenic programming, both in adulthood and development.

Catecholamines, particularly norepinephrine, are central regulators of thermogenesis in BAT and the browning of WAT into beige adipocytes. Sexual dimorphism significantly influences these processes, affecting both the extent and efficacy of catecholamine induced thermogenesis. In rodent models, female mice exhibit a more robust browning response in gonadal WAT compared to males. This heightened response correlates with increased sympathetic innervation, as indicated by elevated tyrosine hydroxylase levels, and higher expression of neurotrophins like nerve growth factor (NGF) and brain derived neurotrophic factor (BDNF) in females [146]. Ovariectomy, which reduces estrogen levels, diminishes these effects, underscoring estrogen’s role in enhancing sympathetic activity and browning capacity in female adipose tissue.

Human studies mirror these findings, revealing that premenopausal women have a higher ratio of α2- to β-adrenergic receptors in subcutaneous fat, leading to reduced catecholamine stimulated lipolysis compared to men and postmenopausal women. This receptor distribution contributes to sex specific differences in fat distribution and metabolic responses [147]. At the molecular level, catecholamine induced activation of β-ARs, which stimulates cyclic adenosine monophosphate (cAMP) production, leads to the activation of protein kinase A (PKA) and subsequent upregulation of UCP1 [148], a key mediator of thermogenesis. Recent research has identified the cAMP-binding protein EPAC1 as a critical regulator of BAT growth and beige adipogenesis. Activation of Exchange Protein directly activated by cyclic AMP 1 (EPAC1) enhances BAT mass and promotes the browning of WAT, contributing to increased energy expenditure and improved metabolic profiles [149].

Furthermore, the interplay between catecholamines and sex hormones extends to the regulation of gene expression involved in adipocyte differentiation and function. For instance, estrogen has been shown to enhance the expression of PGC-1α, a coactivator that drives mitochondrial biogenesis and UCP1 expression, thereby amplifying the thermogenic response [150, 151]. In contrast, androgens may suppress these pathways, leading to diminished thermogenic capacity [152].​ Collectively, these findings highlight the intricate relationship between catecholamines, sex hormones, and adipose tissue function. Understanding the sex specific mechanisms governing BAT activation and WAT browning is crucial for developing targeted therapies for metabolic disorders, taking into account the hormonal milieu and its impact on adipose tissue plasticity.

### Molecular signature of brown and beige adipocytes in males and females

Sex specific molecular differences in brown and beige adipocytes are increasingly recognized as key determinants of thermogenic capacity and metabolic health. Classical brown adipocytes, primarily found in interscapular depots, originate from Myf5+, Pax7+, and Engrailed 1 + dermomyotome lineages [153], and are enriched in brown fat specific markers such as Lhx8, Zic1, Eva1 (Mpzl2), and epithelial-stromal interaction protein 1 (Epsti1) [78]. Beige adipocytes, inducible within white adipose tissue, have distinct developmental origins and gene expression patterns. Non Myf5 + progenitor derived beige cells express markers such as Tmem26, CD137, CD40, and T-box transcription factor 1 (Tbx1), especially under cold exposure or β3-adrenergic stimulation. Transcriptome analyses further reveal beige enriched genes including Kelch-Like Family Member 13 (Klh113), V-erbA-related protein 2 (Ear2), Speckled 100 kDa nuclear autoantigen (Sp100), and solute carrier family 27 A (Slc27a) [51]. In humans, CITED1, HOXC8 and HOXC9 have been proposed as reliable markers of beige adipocytes, particularly in supraclavicular depots [78].

Recent studies underscore sex linked differences in thermogenic gene expression. Human perirenal BAT expresses higher levels of thermogenic regulators such as 3-Hydroxymethylglutaryl-CoA synthase 2 (HMGCS2), Creatine Kinase, Mitochondrial 1 A/1B (CKMT1A/1B), Potassium channel subfamily K member 3 (KCNK3), and PGC1α [154]. Thermogenic effectors including UCP1, acyl-CoA thioesterase 11 (ACOT11), Glycogen Phosphorylase, Muscle Associated (PYGM), and Fatty Acid Binding Protein 3 (FABP3) strongly correlate with BAT metabolic activity [154]. In murine models, sex specific transcriptional signatures are also evident in brown and beige fat. For example, UCP1, UCP2, BMP8b, PGC1α, COX8, cytochrome c oxidase subunit 7A1 (Cox7a1) is higher in female than in male mice following cold exposure or β3-adrenergic stimulation. These responses are particularly pronounced in gonadal white adipose tissue (gWAT), where females show enhanced sympathetic innervation and greater induction of thermogenic genes, including TH, NGF, and BDNF, compared to males [12].

In a recent study, in BAT, maternal high fat diet al.tered the triglyceride profile and transcriptional activity in a sex dependent manner. Female offspring exhibited increased expression of thermogenic and lipid processing genes including UCP1, elongation of very long chain fatty acids-3 (Elovl3), lipoprotein lipase (LPL), Pparα, Pparγ, and Adrenoceptor Beta 3 (Adrβ3), while male offspring showed higher CideA and lower PGC1α and PRDM16, indicating sex specific BAT programming [155]. Notably, BAT lipid remodeling in females occurred with minimal increases in fat mass, suggesting greater metabolic plasticity and preservation of BAT function. This is further supported by recent findings from Paz et al. [156], who demonstrated that maternal high fat diet induced sexually dimorphic alterations in offspring brown adipose tissue. Male offspring exhibited increased lipid accumulation in BAT and upregulated expression of Pparγ and cluster of differentiation 36 (Cd36), whereas females under control diet showed higher expression of fatty acid synthase (Fasn). Maternal HFD reduced carnitine palmitoyltransferase 1 A (Cpt1a) and fatty acid binding protein 4 (Fabp4) expression in female BAT but had no significant effect on these genes in males, indicating a female specific transcriptional sensitivity to early life nutritional programming.

Sexual dimorphism in the molecular expression of brown in white (brite) adipocyte markers reveals differential thermogenic potential between males and females. Servera et al. [157] investigated the inguinal adipose tissue of adult male and female rats and reported that males exhibited significantly higher expression of several brite associated genes, including CideA, homeobox C9 (HOXC9), and short stature homeobox 2 (SHOX2), irrespective of dietary condition or early life leucine exposure. CideEA, a marker of thermogenic competence in multilocular adipocytes, was consistently elevated at both mRNA and protein levels in males compared to females, suggesting a more robust browning capacity in male inguinal WAT depots [157]. Further analysis of metabolic regulators reinforced these findings. Genes associated with lipid metabolism and adipocyte function, leptin (Lep), resistin (RETN), adiponectin (AdipoQ), LPL, adipose triglyceride lipase (ATGL), carnitine palmitoyltransferase 1b muscle isoform (CPT1), fatty acid synthase (FASN), and stearoyl-Coenzyme A desaturase 2 (SCD2), were expressed at significantly higher levels in males. Transcriptional regulators including sterol regulatory element binding transcription factor 1c (SREBP1c) and PPARγ2, both critical for adipocyte differentiation and lipid homeostasis, also displayed male biased expression, particularly under early life leucine supplementation. This dietary intervention upregulated CideA, PPARγ, and LEP, and downregulated CPT1 in both sexes, but the effects were more pronounced in females, indicating sex specific programming of adipose tissue responsiveness.

Although direct sex based comparisons are lacking in current epigenetic literature, several epigenetic regulators have been implicated in the control of brown and beige adipocyte development. Notably, SIRT1, Histone Deacetylase 1 and 3 (HDAC1/3), and the PRDM16 -EHMT1 complex modulate chromatin accessibility at key thermogenic loci such as UCP1 and PGC-1α, influencing BAT differentiation and function [158]. DNA methylation via DNA -methyltransferase 3 alpha (DNMT3a) also suppresses browning of white adipocytes by silencing beiging-related genes [158]. Given that many of these epigenetic regulators are hormonally sensitive, it is plausible that they contribute to sex specific thermogenic regulation; however, sex stratified analyses are urgently needed to validate this hypothesis.

Sex specific molecular and epigenetic differences shape the thermogenic capacity and functional adaptability of brown and beige adipocytes. These differences, influenced by developmental origins, transcriptional profiles, and hormonal sensitivity, contribute to distinct metabolic phenotypes in males and females. Future studies incorporating sex stratified analyses are essential to fully elucidate these mechanisms and their relevance to metabolic health.

## Brown Fat, sexual Dimorphism, and disease

BAT has emerged as a key modulator of energy metabolism, with its activity closely linked to protection against obesity and metabolic diseases. Notably, sexual dimorphism in BAT function contributes to distinct patterns of fat distribution, metabolic flexibility, and disease susceptibility between males and females. These differences become particularly relevant in the context of aging and hormonal changes, influencing the onset and progression of metabolic disorders. Understanding the interplay between BAT/beige, sex hormones, and disease risk offers promising avenues for developing gender specific therapeutic strategies targeting metabolic health.

### Sex differences in obesity and metabolic disorders

Epidemiological evidence shows that although women have higher overall obesity rates, men are more prone to visceral fat accumulation, which confers greater metabolic risk [159]. The composition of metabolic risk factors varies by sex and geography [160]. In the U.S., between 1999 and 2000, metabolic risk prevalence rose significantly in women aged 20–39 years compared to men, driven largely by rising obesity rates in women [161]. This disparity was primarily driven by a higher increase in obesity rates in women, with 2 million more women than men affected in the U.S. at the time of analysis. Similarly, in Mauritius, non-diabetic women had higher BMIs and post glucose load values than men, suggesting greater glucose intolerance susceptibility [162]. Before menopause, women tend to accumulate subcutaneous fat, while men and postmenopausal women preferentially store visceral fat, increasing their risk for insulin resistance and cardiovascular disease [160].

A German study found undiagnosed diabetes was more common in men (9.3%) than women (6.9%), yet women were more frequently diagnosed with impaired glucose tolerance via 2 h glucose challenge, revealing a diagnostic gap when relying on fasting glucose alone [160]. Inflammation, assessed by CRP levels, was a stronger predictor of metabolic syndrome in women than men [163]. Visceral and android fat, more prevalent in men elevate cardiometabolic risk, and genetic factors like PPARγ polymorphisms may differentially influence obesity traits between sexes [160]. These findings underscore the importance of sex specific screening and therapeutic strategies for obesity and metabolic disorders.

Building on these epidemiological patterns, recent evidence highlights the role of BAT in modulating sex specific susceptibility to cardiometabolic disorders. A large retrospective analysis of PET-CT scans from 52,487 patients revealed significant associations between BAT presence and improved cardiometabolic health, with notable sex based differences. Women exhibited a significantly higher prevalence of detectable BAT than men (13.8% vs. 4.9%, *P* < 0.0001), aligning with prior findings that BAT activity is more prominent in females [164]. This discrepancy in BAT prevalence may offer a physiological explanation for the observed sex based resilience to cardiometabolic diseases, despite higher overall obesity rates in women. While men are more prone to visceral fat accumulation and associated risks, the thermogenic and endocrine functions of BAT may offset these disadvantages in women.

Importantly, individuals with BAT had markedly lower prevalence of T2DM (4.6% vs. 9.5%), dyslipidemia (18.9% vs. 22.2%), coronary artery disease (3.1% vs. 4.9%), cerebrovascular disease (2.1% vs. 2.8%), congestive heart failure (1.0% vs. 2.0%), and hypertension (26.7% vs. 30.7%), all with *P* < 0.01 significance compared to BAT negative individuals [164]. Multivariable logistic regression analysis confirmed BAT as an independent negative predictor of these conditions, with adjusted odds ratios ranging from 0.44 for T2DM to 0.85 for hypertension. Notably, the protective effect of BAT was most pronounced in individuals with obesity; for instance, obese BAT positive individuals had a T2DM prevalence of 7.5% compared to 20.3% in their BAT negative counterparts (*P* < 0.0001) [164]. Thus, despite a higher prevalence of obesity in women, the greater abundance of thermogenically active BAT may confer protection against metabolic disorders, emphasizing the need to account for adipose tissue phenotype in sex specific assessments of cardiometabolic risk.

Furthermore, in conditions such as PCOS, a prevalent endocrine metabolic disorder in women, is closely associated with adipose tissue dysfunction. Although traditionally focused on WAT, recent evidence implicates BAT in the pathophysiology of PCOS. Women with PCOS exhibit lower supraclavicular skin temperature and BAT volume, indicative of impaired thermogenesis. BAT volume is also decreased in PCOS [125], with studies reporting a negative correlation between BAT mass and BMI in affected individuals. This dysfunction is linked to elevated androgen levels, which suppress UCP1 expression and mitochondrial respiration in brown adipocytes. This androgen induced BAT dysfunction may exacerbate insulin resistance and central adiposity, key features of PCOS. These findings suggest that elevated androgens may drive sex specific differences in BAT activity and contribute to the metabolic dysfunction characteristic of PCOS.​ [165].

### BAT in age related and hormonal disorders

BAT mass and activity decline with age in both sexes, but women retain greater BAT volume and function across the lifespan. Although BAT levels decline with age in both sexes, women consistently exhibit higher BAT levels than men, especially during youth, and maintain superior BAT activity and non-shivering thermogenesis into adulthood [8, 101]. In rodents, aging males show reduced BAT mass and thermogenic markers, while females retain BAT, indicating a slower decline [166, 167]. This preservation may stem from enhanced sympathetic innervation in females, evidenced by higher tyrosine hydroxylase and β3-adrenergic receptor expression in WAT, supporting greater browning and thermogenic output [146]. Further to validate, in a study involving 162 healthy adults aged 20–73 years, the incidence of cold activated BAT decreased from over 50% in individuals in their twenties to less than 10% in those in their fifties and sixties. This decline correlated with increased body fat accumulation and was more pronounced in men. Conversely, women consistently displayed higher BAT activity and mass with age compared to men, reinforcing sex differences in BAT aging [168].

The menopausal transition is marked by a dramatic reduction in estrogen levels, which plays a critical role in maintaining BAT thermogenesis. In a clinical study, Blondin et al. [109] reported that premenopausal women had significantly higher cold stimulated BAT oxidative metabolism (1.28 ± 0.85 vs. 0.91 ± 0.63 min⁻¹) and glucose uptake compared to postmenopausal women. Pharmacological suppression of ovarian function in premenopausal women mirrored this reduction, confirming that estrogen plays a direct role in regulating BAT activity independent of age or thermoregulatory demand [109]. Estrogen decline is also associated with a shift from subcutaneous to visceral adiposity, increasing visceral fat from 5 to 8% to 15–20% of total fat mass after menopause, thereby raising cardiometabolic risk [169], predisposing postmenopausal women to insulin resistance, dyslipidemia, and systemic inflammation, thereby increasing the risk of type 2 diabetes and cardiovascular disease [170].​

In aging men, a gradual decline in testosterone, termed andropause, is linked to increased fat mass and reduced lean mass. Lantero Rodriguez et al. [171] demonstrated that testosterone suppresses BAT metabolic activity in male mice: castration induced testosterone deficiency enhanced BAT activity, whereas testosterone replacement reduced it [171]. These findings suggest that declining testosterone levels with age may influence BAT function and contribute to metabolic changes observed in aging men.

Additionally, GC modulate BAT function and are altered with age. Short term administration of prednisolone in healthy young men increased cold induced ^18F-FDG uptake in BAT, elevated supraclavicular skin temperature, and enhanced energy expenditure during cold exposure [133]. In vitro, cortisol acutely enhanced UCP1 expression and thermogenic respiration in human BAT cells, but chronic or high dose exposure blunted UCP1 levels, revealing a biphasic effect. A retrospective PET/CT study further showed that individuals on chronic GC therapy had significantly reduced BAT volume and activity compared to controls [133]. Collectively, these findings underscore that age related hormonal shifts, including the loss of estrogen, testosterone decline, and altered glucocorticoid exposure, can disrupt thermogenic regulation via BAT, contributing to age associated metabolic dysfunction. Understanding these endocrine BAT interactions is crucial for developing sex specific interventions targeting energy balance and metabolic health.

### Potential for BAT activation in sex specific therapeutics

BAT has emerged as a critical player in systemic energy homeostasis, with significant implications for managing metabolic diseases and promoting healthy aging. Given its sexually dimorphic distribution and responsiveness to hormones, targeted activation of BAT represents a promising therapeutic avenue, especially when guided by sex specific biological insights.

Evidence from long lived genetic mouse models suggests a strong link between enhanced BAT activity and improved metabolic health. Overexpression of the tumor suppressor gene phosphatase and tensin homolog (Pten) increases lifespan, accompanied by elevated BAT activity, enhanced energy expenditure, and reduced obesity in mice [172]. Interestingly, sex specific effects have been observed with FGF21 overexpression: female mice experience more robust lifespan extension than males, potentially due to their greater propensity for WAT browning and improved metabolic flexibility [173]. Growth hormone (GH) signaling also modulates BAT function. Ames dwarf mice (which lack GH production) and growth hormone receptor (GHR) knockout mice show increased BAT mass and activity, as well as transcriptomic differences between sexes within BAT [174, 175]. Recent transcriptomic analyses of BAT from long lived GHRH knockout mice have further highlighted sex specific regulatory patterns in response to GH downregulation [174]. While both sexes show metabolic benefits and extended lifespan, male GH deficient mice appear to be more susceptible to certain stressors such as paraquat exposure, compared to their female counterparts [176]. This suggests that BAT adapts differently in males and females under reduced GH signaling. In humans, the picture is more complex. While lower GH levels have been linked to adverse outcomes like reduced bone density, altered cardiac function, and increased risk of nonalcoholic fatty liver disease [177], individuals with Laron Syndrome, a condition caused by mutations in the GHR gene, share several traits with GHR deficient mice, including short stature and increased fat mass. Interestingly, they are also largely protected from age related diseases such as cancer [178]. Whether these individuals exhibit increased BAT activity or show sex specific thermogenic responses remains unclear.

Caloric restriction (CR) remains the most consistent non genetic intervention to extend lifespan across species. In rats, CR enhances mitochondrial function in BAT of both sexes but appears to produce more pronounced molecular remodeling in females. Valle et al. [179] showed that old female rats under CR displayed lower BAT mass and reduced UCP1 expression, suggesting slowed lipid accumulation and preserved thermogenic efficiency relative to males. These changes may underlie stronger metabolic resilience in females under nutrient scarcity. Ketogenic diets and protein restriction also influence BAT activity, primarily via endocrine mediators such as FGF21. In male rodents, low protein diets have been shown to increase BAT sympathetic innervation and thermogenesis, potentially through FGF21 upregulation [180, 181]. However, female specific data are scarce, underscoring the need for sex balanced studies.

Conversely, Western diets exacerbate adipose inflammation and metabolic dysfunction with age. Yet, female mice show a degree of protection against obesity induced inflammation. In a diet induced obesity model, Varghese et al. [182] found that older obese females, despite increased visceral fat, maintained higher expression of oxidative metabolism markers like Pparγ and Pgc1α, and showed an attenuated inflammatory response in visceral WAT. In contrast, older obese males exhibited elevated integrin, alpha X (CD11c⁺) proinflammatory macrophages and higher circulating insulin levels, reflecting more severe metabolic impairment. Interestingly, stimulated lipolysis in females did not worsen inflammation, suggesting a preserved adaptability in adipose tissue remodeling. These findings highlight the potential for sex specific BAT or WAT targeted nutritional interventions in aging populations.

Several pharmacological agents with longevity benefits may exert part of their action through BAT. Metformin, a first line antidiabetic drug, accumulates in BAT and has been shown to increase BAT thermogenesis and oxygen consumption in mice [183]. However, its sex specific efficacy remains controversial. While some studies report beneficial effects in female mice [184] and male mice [185], others found neutral or even negative effects, possibly due to strain or dose differences [186, 187]. Resveratrol, a polyphenol with potential anti-aging effects, promotes browning of WAT via UCP1 upregulation and PGC-1α activation. In rats, resveratrol enhances mitochondrial biogenesis in BAT, though sex specific responses have not been thoroughly explored [188]. Sirtuin 1 activation, a known target of resveratrol, has been implicated in restoring age related mitochondrial decline in BAT [189], but whether this effect varies by sex remains unknown. 17α-estradiol, an estrogen receptor agonist, extends lifespan in males (19%) with no significant effect on female lifespan [190], likely via endocrine modulation. Although BAT size remained unchanged, 17α-estradiol treated males exhibited improved metabolic outcomes, including reduced adiposity, enhanced insulin sensitivity, and decreased systemic inflammation, indicating a shift toward greater metabolic efficiency without changes in BAT size [191]. Collectively, these findings highlights that BAT targeted interventions, whether genetic, dietary, or pharmacological, may yield different outcomes depending on sex.

### Endocrine disrupting chemicals and sexual dimorphism in BAT/Beige biology

The U.S. Environmental Protection Agency (EPA) describes an endocrine disrupting chemicals (EDCs) as an external substance that alters the normal synthesis, secretion, transport, metabolism, binding, action, or clearance of natural hormones in the bloodstream, hormones that are essential for maintaining homeostasis, reproduction, and developmental processes [192]. Common examples include polychlorinated biphenyls (PCBs), polybrominated biphenyls (PBBs), dioxins, bisphenol A (BPA), phthalates, pesticides, and dichlorodiphenyltrichloroethane (DDT). EDCs are of particular concern in the context of adipose tissue because they often mimic or antagonize the actions of endogenous hormones, including estrogens, androgens, thyroid hormones, and glucocorticoids [193], all of which are central to brown and beige adipocyte development and thermogenic function. Experimental studies show that EDCs such as BPA accumulate in BAT and produce sex specific effects, enhancing thermogenic gene expression in females while impairing brown adipogenesis in males [193]. These findings indicate that sex may shape the impact of EDCs on brown and beige fat, with implications for metabolic risk.

In female rats, BPA accumulates in BAT at concentrations markedly higher than in blood, brain, or WAT, suggesting that BAT is a specific site of vulnerability to lipophilic endocrine disruptors [194]. Developmental exposure further revealed sex dependent outcomes: gestational BPA increased interscapular BAT mass and upregulated Ucp1 expression in female offspring, whereas male offspring displayed reduced brown adipogenesis and impaired BAT activity [195]. Mechanistically, in females, BPA can act through estrogen receptors, which are essential for mitochondrial biogenesis and thermogenesis in BAT [196]. In males, detrimental effects are linked to disruption of thyroid hormone signaling. BAT relies on local T_3_ production by DIO_2_ to drive Ucp1 expression and support WAT beiging [193]. Ex vivo studies of male BAT demonstrated that BPA reduces DIO_2_ activity in a concentration dependent manner [197], providing evidence for thyroid related impairment.

Other EDC classes also show direct actions on thermogenic adipose tissue. Radiolabeled diethyl-hexylphthalate (DEHP) (a common phthalate) showed high initial uptake into BAT, with persistent radioactivity after 24 h, indicating that BAT is a major target depot [198]. Developmental exposure produced hyperplastic BAT, characterized by larger scapular depots and greater adipocyte numbers [199]. In contrast, short term adult exposure reduced Ucp1 expression, induced hypothermia and hyperphagia, and promoted weight gain along with gut dysbiosis in male mice [200, 201]. These stage and sex dependent effects suggest that phthalates disrupt thermogenic programming differently across life stages.

Perinatal exposure to organochlorines further underscores sex specific susceptibility. In a murine model, DDT exposure impaired metabolic homeostasis in females but not males, with early life weight gain attributed to BAT dysfunction and reduced energy expenditure. Over time, this progressed to insulin resistance and metabolic syndrome under high fat diet challenge [202].

Evidence from human cohorts also supports sex biased sensitivity. Persistent organic pollutants (POPs) in WAT were associated with adipocyte hypertrophy, macrophage infiltration, systemic inflammation, and impaired glucose metabolism, with these effects generally stronger in women [202]. Correlations between circulating PCB levels and leptin were significant in females but not males, indicating sex differential endocrine disruption in adipose tissue [203]. Although these studies did not directly measure BAT, they reinforce the principle that pollutant hormone interactions in adipose biology are sex contingent.

Taken together, these findings demonstrate that EDCs disrupt thermogenic adipose tissue in a manner that is strongly influenced by sex. By mimicking estrogenic activity, BPA enhances BAT development and Ucp1 expression in females, while simultaneously impairing thyroid driven thermogenesis in males. Similar sex and stage dependent patterns are evident with phthalates, organochlorines, and persistent organic pollutants, underscoring that endocrine disruption intersects with the intrinsic sexual dimorphism of brown and beige fat. Recognizing these interactions is essential for understanding how environmental exposures contribute to sex specific risk of obesity and metabolic disease.

## Challenges and future directions

Despite growing research interest, key gaps remain in the field. Most notably, large scale, sex disaggregated clinical studies are lacking, limiting the translation of findings from animal models to humans. Challenges persist in accurately quantifying human BAT activity. ^18F-FDG PET/CT remains the gold standard for assessing BAT, but it underestimates total BAT volume and is insensitive to lipid oxidation, which is critical for thermogenic output [204]. Emerging imaging tools, such as infrared thermography and novel radiotracers like ^11^C acetate and ^18^F-FTHA, offer promise in capturing sex specific BAT metabolism more precisely.

BAT function is regulated by a complex endocrine network extending beyond sex steroids. Numerous hormones, including thyroid hormones, catecholamines, growth hormone, IGF-1, prolactin, insulin, and endocannabinoids, modulate BAT recruitment and thermogenesis [205]. However, the sex specific roles of these hormones in BAT regulation remain poorly characterized and require targeted investigation. Moreover, disruption of hormonal homeostasis across the lifespan, such as estrogen decline in menopause, testosterone loss in andropause, and chronic glucocorticoid exposure, has been shown to impair BAT function and thermogenic capacity, exacerbating age related adiposity and metabolic dysfunction.

Epigenetic regulation also plays a critical role in modulating BAT development and responsiveness to environmental and hormonal signals. Mechanisms involving DNA methylation and histone modifications influence UCP1 expression and beiging capacity, often in a sex dependent manner. Estrogen receptors α and β have been implicated in DNA demethylation and WAT browning [206], while environmental cues, such as paternal undernutrition, have been shown to program offspring BAT through sperm methylation patterns [207]. Understanding how these sex specific epigenetic mechanisms interact with endocrine signals may pave the way for personalized metabolic therapies.

Environmental exposures further complicate thermogenic adipose regulation. Animal studies clearly show that EDCs, including BPA and phthalates, act in a sex dependent manner enhancing thermogenesis in females while suppressing BAT function in males. Yet whether these dimorphic effects translate to humans remains uncertain. Current tools for assessing BAT activity in exposed populations are limited, and clinical studies have not systematically accounted for sex differences. Moving forward, regulatory and research frameworks will need to explicitly address sex specific susceptibility to environmental disruption to better understand and mitigate the metabolic consequences of EDC exposure. In parallel, pharmacological approaches targeting BAT have gained traction, yet sex specific responses remain underexplored. BAT targeted pharmacology is gaining traction, yet sex effects are underexplored; pharmaceuticals such as metformin enhance BAT activity in rodents [208], while 17α-estradiol confers metabolic and longevity benefits predominantly in males [191]. Nutraceuticals like resveratrol can also activate BAT, with responses across these interventions varying by sex and genetic background [209, 210]. While rapamycin improves lifespan, it paradoxically impairs WAT browning and induces cold intolerance [211]. These findings reinforce the importance of delineating how sex modulates the efficacy and adverse effects of BAT targeted therapies.

Despite promising advances, a major bottleneck in clinical translation is the lack of reliable, non-invasive tools for longitudinal BAT assessment in humans and the incomplete understanding of BAT’s integration into whole body energy regulation across sexes. To bridge this gap, future research must prioritize sex stratified studies using advanced imaging, molecular profiling, and computational modeling. Integrating these tools with personalized medicine frameworks will enable the design of tailored BAT based interventions for obesity and metabolic diseases, accounting for individual hormonal, genetic, and epigenetic profiles.

## Conclusion

Considering sex as a key biological factor is essential for understanding brown and beige adipose tissue biology and its therapeutic potential. This review highlights that BAT is not only anatomically and functionally distinct between males and females, but its recruitment, activation, and age associated decline are tightly regulated by sex hormones and their downstream signaling cascades. Estrogens promote BAT activity and WAT beiging via ERα mediated mechanisms, whereas testosterone and glucocorticoids generally suppress thermogenic programming, contributing to sex specific vulnerabilities in metabolic disease. Sexual dimorphism is further amplified across the lifespan, with menopause and andropause triggering differential declines in thermogenic capacity and shifts in fat distribution. These hormonally driven changes significantly influence disease susceptibility, with diminished BAT activity implicated in the onset of insulin resistance, visceral adiposity, and cardiometabolic dysfunction, especially in aging populations.

Emerging evidence from genetic, dietary, and pharmacological interventions suggests that enhancing BAT activity holds promise in promoting healthy aging and combating obesity related disorders. However, most studies remain male biased or neglect sex stratified analysis, limiting translational insight. The influence of environmental disruptors, epigenetic reprogramming, and hormonal modulators on BAT function also exhibits sex dependent patterns, underscoring the need for more nuanced research models. Future therapeutic strategies should move beyond one size fits all approaches and embrace sex informed interventions that harness BAT plasticity and endocrine responsiveness. Advancing BAT based treatments will demand a more integrated research approach combining molecular profiling, advanced imaging, and systems biology to define individualized metabolic phenotypes. Only through such personalized frameworks can the full potential of BAT be realized in the context of obesity, aging, and metabolic disease.

## Data Availability

No datasets were generated or analysed during the current study.
